# Early motor development: risk factors for delay in a population study in Southern Brazil

**DOI:** 10.11606/s1518-8787.2023057004991

**Published:** 2023-09-14

**Authors:** Ana Carolina Zago, Jéssica Puchalski Trettim, Bárbara Borges Rubin, Carolina Coelho Scholl, Fernanda Teixeira Coelho, Fernanda Ulguim, Luísa Mendonça de Souza Pinheiro, Mariana Bonati de Matos, Ricardo Tavares Pinheiro, Luciana de Avila Quevedo

**Affiliations:** I Universidade Católica de Pelotas Programa de Pós-Graduação em Saúde e Comportamento Pelotas RS Brasil Universidade Católica de Pelotas. Programa de Pós-Graduação em Saúde e Comportamento. Pelotas, RS, Brasil.; II Centro Universitário da Região da Campanha Bagé RS Brasil Centro Universitário da Região da Campanha. Bagé, RS, Brasil.

**Keywords:** Child Development, Infant, Low Birth Weight, Cesarean Section

## Abstract

**OBJECTIVE:**

To assess risk factors associated with motor development delay at three months of age.

**METHODS:**

Cross-sectional study with mothers and their three-month-old babies in Southern Brazil. The Bayley-III Scale of Infant and Toddler Development (BSID-III) and the Alberta Infant Motor Scale (AIMS) were used to assess motor development.

**RESULTS:**

We evaluated 756 mothers and their three-month-old babies. The overall mean motor development assessed by the BSID-III and the AIMS was 104.7 (SD 13.5) and 55.4 (SD 25.4), respectively. When assessed by the BSID-III, the lowest motor development scores were among babies born by cesarean delivery (p = 0.002), prematurely (p < 0.001), and with low birth weight (p < 0.001). When assessed by the AIMS, babies born prematurely (p = 0.002) and with low birth weight (p=0.004) had the lowest motor development means. After a cluster analysis, we found that babies born by cesarean delivery, with low birth weight, and prematurely had more impaired motor development compared with children born without any risk factors.

**CONCLUSION:**

Identifying risk factors allows the implementation of early interventions to prevent motor development delay and, therefore, reduce the probability of other future problems.

## INTRODUCTION

Child development is a process that begins during pregnancy and involves several factors, such as physical growth, neurological maturation, and the building of skills related to behavior and the cognitive, social, and affective spheres^1^. During the first years of life, motor skills increases greatly. Regarding motor development, the evolution occurs in a craniocaudal manner, with the acquisition of cervical tone at around three months of age, followed by the ability to seat at six months, and the ability to stand at around 12 months of age. In early life, movement variation serves for exploration, followed by trial an error^2^.

Current literature considers motor development a non-linear process with transition phases, which are affected by many risk factors. These factors include child and birth-related characteristics, such as being born prematurely and/or with low birth weight, being a boy, and having fewer siblings^3^, and environmental characteristics, such as lower maternal age, lower maternal schooling, and substance abuse (alcohol and tobacco) during pregnancy^9^. On the other hand, studies point to some protective factors that contribute to a better motor development of the baby, such as breastfeeding and vaginal delivery^12^.

Delays in child development in different age groups are strongly associated with premature birth and, consequently, with low birth weight^15,16^. Most research on child development that focus on motor skills comes from developed countries and uses samples with children at older ages. Therefore, assessing early motor development in developing countries is also essential, so that appropriate interventions can be adopted when necessary, ensuring that children have a better development throughout their life and preventing further damage.

From this perspective, technological advances in the care of premature newborns have contributed to the reduction of mortality levels in this population. However, the number of premature babies with alterations in neuromotor function, hearing, language, and cognitive development has increased^17^. A better understanding of these changes is extremely important, since premature babies may have negative effects in psychomotor skills and impaired learning when they reach school age. Early diagnosis of these alterations is essential so that interventions on child development begin as early as possible^18^.

Besides prematurity, low birth weight (LBW) is a factor that may be related to impaired child development. The World Health Organization (WHO) defines LBW as a birth weight below 2,500 grams, regardless of the gestational period. LBW is a determinant factor of malnutrition, influencing child growth and development, and, in the long term, may affect health conditions in adulthood^19^. Moreover, alcohol and tobacco use during pregnancy^20,21^, cardiovascular, respiratory, neurological disorders, neonatal infections, malnutrition, low socioeconomic conditions, and poor parental schooling are the main causes for motor development delay^22,23^.

Identifying risk factors for delayed motor development in early childhood is essential for planning interventions, counseling parents, and updating and specifying information for professionals about children at early ages. Therefore, this study aimed to assess risk factors, such as sociodemographic, maternal, behavioral, gestational, perinatal, and child-related variables, for motor development delay in the first three months of a child’s life.

## METHODS

This population-based cross-sectional study was performed with pregnant women in the urban area of the city of Pelotas, Rio Grande do Sul, Brazil. The sampling process included multiple stages, with the census sectors delimited by the *Instituto Brasileiro de Geografia e Estatística* (Brazilian Institute of Statistics - IBGE) as primary sampling units. For more details, see Pinheiro et al.^24^. From May 2016 to August 2018, the research team invited women up to 24 weeks pregnant to participate in the study. After this step and 90 days after delivery, these women and their children were assessed. This assessment took place at the institution where the study was conducted, in a structured, standardized, and stimulus-neutral room, so that the babies could be assessed by trained psychologists and physical therapists. The duration of the motor development assessment was about 30 minutes. During the baby’s assessment, the mother was in the same room, answering the study interview and available to attend to the baby’s needs, such as breastfeeding and sleeping.

In order to assess motor development at three months of age, the motor subscale of the Bayley-III Scale of Infant and Toddler Development (BSID-III) was applied. This is considered the “gold standard” tool for the assessment of child development. The BSID-III is individually administered and assesses motor development by the observation of changes in the child’s posture and behavior in response to standardized stimuli. The BSID-III has no validated cut-off point for the Brazilian population. Thus, for the analysis in this study, the composite score was used, which is calculated by the weighted score according to the child’s age corrected for prematurity. Higher scores show better motor development^25,26^.

Since the BSID-III has no validated cut-off point for the Brazilian population, the Alberta Infant Motor Scale (AIMS), which is validated for this population^27^, was also used. The AIMS is an observational scale for the assessment of broad motor development and includes 58 items that analyzes children’s spontaneous movement in four subscales (or postures): prone positioning (21 items), supine positioning (nine items), sitting (12 items), and standing (16 items). The raw score is obtained by summing the points from the four subscales. Based on this score, and considering the corrected age of the child, the gross motor performance percentile is identified^28^.

The socioeconomic classification used the criteria of the *Associação Brasileira de Empresas de Pesquisa* (Brazilian Association of Research Companies - ABEP), which is based on the accumulation of material goods, the schooling level of the head of household, and whether the residence has piped water and paved street. This classification divides the participants into five levels (A, B, C, D, or E), based on the scores achieved. The letter “A” refers to the highest socioeconomic level and the letter “E” to the lowest^29^. For this study, the levels were combined: high (A+B), medium (C), and low (D+E).

Moreover, the following maternal variables were assessed: age (up to 23 years old; 24–29 years old; 30 years old or more), schooling (up to eight full years of study; nine full years of study or more), living with a partner (no/yes), psychiatric drug use during pregnancy (no/yes), and alcohol and tobacco use during pregnancy (no/yes). The following gestational and baby variables were also considered: first pregnancy (no/yes), gestational hypertension (no/yes), gestational diabetes (no/yes), type of delivery (vaginal/cesarean), baby’s sex (boy/girl), prematurity (up to 36 weeks and six days), low birth weight (2,499 g or less), and siblings (no/yes). The variable “psychological and/or drug treatment” was included for adjustment purposes as a confounding factor, considering its possible effect on motor development.

After encoding the tools, data were double entered into EpiData 3.1 to test their consistency. For data analysis, SPSS software version 24.0 was used. In univariate analysis, simple and relative frequency, mean, and standard deviation were used to describe the characteristics of the sample. In order to compare the means between motor development and exposure variables, the analysis of variance (ANOVA) test was used. For the adjusted analysis, all variables with p < 0.20 were included in a linear regression. A cluster analysis was performed for the BSID-III (the gold standard for assessing motor development), using ANOVA and Tukey’s test. Thus, the following categories were created: 1) none of the perinatal risk factors; 2) cesarean delivery as the only risk factor; 3) cesarean delivery and low birth weight as risk factors; 4) cesarean delivery and prematurity as risk factors; and 5) three risk factors (cesarean delivery, low birth weight, and prematurity).

To verify multicollinearity in the regression analyses between the variables that remained in the regression model, the variance inflation factor (VIF) and tolerance were estimated. A VIF above 4 or a tolerance below 0.25 points the existence of multicollinearity between the variables. All participants signed an informed consent form, allowing them and their babies to participate. The research team advised the mothers on activities to stimulate the baby’s development.

## RESULTS

We evaluated 756 mothers and their three-months-old babies. Table 1 shows the sample distribution. Of the 981 pregnant women who participated in the first assessment of the study, 756 (77.1%) returned 90 days after delivery. Thus, the loss/refusal rate was 22.9%. Among the losses, 43 (4.4%) were due to miscarriage.

**Table 1:** Sample characteristics and their association with motor development in three-month-old babies, Southern Brazil.

Variables	n (%)	Three-month-old baby motor development			

		BSID-III	p-value	AIMS	p-value
	
		Mean (SD)		Mean (SD)	
Sociodemographic variables					
Maternal age			0.042		0.036
Up to 23 years old	233 (30.8)	106.1 (13.9)		57.7 (26.8)	
24 to 29 years old	249 (32.9)	104.9 (12.9)		56.8 (24.9)	
30 years old or more	274 (36.2)	103.2 (13.4)		52.3 (24.4)	
Socioeconomic status*			0.076		0.064
High (A+B)	203 (27.4)	102.9 (13.9)		53.1 (25.8)	
Medium (C)	412 (55.6)	105.4 (13.5)		55.7 (24.8)	
Low (D+E)	126 (17)	105.7 (12.8)		59.8 (25.7)	
Maternal schooling (full years of study)			0.649		0.084
9 years or more	524 (69.3)	104.8 (13.4)		54.4 (24.5)	
Up to 8 years	232 (30.7)	104.3 (13.6)		57.8 (27.2)	
Maternal behavioral variables					
Living with a partner*			0.665		0.138
No	112 (14.9)	105.2 (12.9)		58.8 (26.5)	
Yes	642 (85.1)	104.6 (13.6)		54.9 (25.2)	
Psychiatric drug use during pregnancy			0.288		0.467
No	739 (97.8)	104.8 (13.5)		55.5 (25.5)	
Yes	17 (2.2)	101.2 (13.5)		51.0 (22.3)	
Alcohol use during pregnancy*			0.781		0.298
No	492 (80.5)	105.2 (13.7)		57.4 (25.2)	
Yes	119 (19.5)	104.8 (13.0)		54.7 (25.4)	
Tobacco use during pregnancy*			0.279		0.863
No	518 (84.8)	105.4 (13.7)		56.9 (25.3)	
Yes	93 (15.2)	103.4 (12.6)		56.4 (25.0)	
Gestational and perinatal variables					
First pregnancy			0.004		0.004
No	437 (57.8)	103.5 (13.5)		53.1 (25.5)	
Yes	319 (42.2)	106.3 (13.3)		58.6 (25.0)	
Psychological and/or drug treatment			0.378		0.475
No	441 (58.3)	104.3 (13.2)		56.0 (25.2)	
Yes	315 (41.7)	105.2 (13.8)		54.6 (25.7)	
Gestational hypertension			0.090		0.311
No	675 (89.3)	104.9 (13,3)		55.7 (25.5)	
Yes	81 (10.7)	102.3 (14.7)		52.7 (24.4)	
Gestational diabetes			0.272		0.917
No	717 (94.8)	104.8 (13.4)		55.4 (25.6)	
Yes	39 (5.2)	102.3 (14.2)		55.8 (21.8)	
Type of delivery*			< 0.001		0.049
Vaginal	269 (36)	107.0 (12.4)		57.9 (25.3)	
Cesarean	478 (64)	103.4 (13.9)		54.1 (25.4)	
Baby variables					
Sex			0.643		0.744
Boy	361 (47.8)	104.4 (12.6)		55.1 (25.6)	
Girl	395 (52.2)	104.9 (14.2)		55.7 (25.3)	
Prematurity*			< 0.001		< 0.001
No	661 (88.4)	106.2 (12.8)		57.6 (24.6)	
Yes	87 (11.6)	93.5 (13.9)		39.5 (26.4)	
Low birth weight*			< 0.001		< 0.001
No	686 (91.0)	105.9 (12.7)		57.1 (24.7)	
Yes	68 (9.0)	92.6 (15.2)		38.1 (26.6)	
Siblings			0.002		0.001
No	300 (39.7)	106.5 (13.0)		59.1 (24.5)	
Yes	456 (60.3)	103.5 (13.6)		53.0 (25.7)	
Total	756 (100.0)	104.7 (13.5)		55.4 (25.4)	

* Variables with missing data.

BSID-III: Bayley-III Scale of Infant and Toddler Development; AIMS: Alberta Infant Motor Scale; SD: standard deviation.

The lowest means for motor development at three months of age were among children of older and multiparous mothers, who were born by cesarean delivery, prematurely, with low birth weight, and had siblings, regardless of the tool used (p < 0.05). We included other variables in the multivariate analysis, since they showed p < 0.20 in the bivariate analysis: socioeconomic status (both tools), maternal age, and living with a partner (AIMS).

After the adjusted analysis, the lowest means for motor development at three months of age assessed by the BSID-III remained associated with cesarean delivery (p = 0.002), prematurity (p < 0.001), and low birth weight (p < 0.001). Children born by cesarean delivery scored 3.1 points (95%CI: −5.0 to −1.1) lower in the mean of the BSID-III compared with babies born by vaginal delivery. Premature babies scored 8.8 points (95%CI: −12.4 to −5.3) lower in the mean of the BSID-III compared with term children. Babies born with low birth weight scored 7.1 points (95%CI: −11.0 to −3.2) lower in the mean of the BSID-III compared with newborns with normal birth weight. Having siblings showed a tendency to impair motor development (p = 0.051). Babies with siblings scored 2.6 points lower in the mean of the BSID-III compared with only children (Table 2).

**Table 2:** Linear regression for exposure variables with motor development at three months of age using the BSID-III.

Exposure variables	Motor development at three months of age (BSID-III)			*t*	VIF

	B	95%CI	p-value		
Maternal age (30 years old or more*)	0.2	−1.1 to 1.5	0.801	0.729	1,372
Socioeconomic status (A+B*)	1.3	−0.3 to 2.8	0.106	0.823	1,215
Primiparity (yes*)	−1.0	−3.4 to 1.7	0.523	0.521	1,921
Gestational hypertension (no*)	−2.1	−5.1 to 1.0	0.180	0.945	1,058
Type of delivery (vaginal*)	−3.0	−5.0 to −1.0	0.004	0.938	1,067
Prematurity (no*)	−8.7	−12.2 to −5.1	< 0.001	0.667	1,498
Low birth weight (no*)	−7.3	−11.2 to −3.3	< 0.001	0.663	1,508
Siblings (no*)	−2.4	−5.1 to 0.1	0.066	0.517	1,933
Psychological and/or drug treatment (no*)	1.5	−0.3 to 3.4	0.109	0.970	1,031

* Reference category.

BSID-III: Bayley-III Scale of Infant and Toddler Development; 95%CI: 95% confidence interval; *t*: tolerance; VIF: variance inflation factor.

Table 3 shows the adjusted analysis regarding the assessment of motor development using the AIMS. The lowest means for motor development remained associated with prematurity (p = 0.002) and low birth weight (p = 0.004). Premature babies scored 11.1 points (95%CI: −18.0 to −4.2) lower in the mean of the AIMS than term newborns. Babies born with low birth weight scored 11.3 points (95%CI: −18.9 to −3.7) lower in the mean of the AIMS than children born with normal weight. Similarly to motor development measured by the BSID-III, having siblings tended to be associated with motor development in the AIMS (p = 0.051). Babies with siblings scored 5.0 points (95%CI: −10.0 to 3.0) lower in the mean of the AIMS compared with only children. The variable maternal psychological and/or drug treatment was a possible confounding factor, showing no association with the outcome, measured by both the BSID-III (Table 2) and the AIMS (Table 3) (p > 0.05).

**Table 3:** Linear regression for exposure variables with motor development at three months of age using the AIMS.

Exposure variables	Motor development at three months of age (AIMS)			*t*	VIF

	B	95%CI	p-value		
Maternal age (30 years old or more*)	0.3	−2.2 to2.9	0.796	0.717	1.395
Socioeconomic status (higher*)	2.0	−1.2 to 5.2	0.213	0.716	1.397
Maternal education (9 years or more*)	3.7	−0.6 to 8.1	0.094	0.796	1.256
Living with a partner (yes*)	−2.2	−7.1 to 2.8	0.386	0.930	1.075
Primiparity (yes*)	−2.1	−7.2 to 2.9	0.404	0.517	1.935
Type of delivery (vaginal*)	−2.3	−6.1 to 1.5	0.240	0.943	1.061
Prematurity (no*)	−11.1	−18.0 to −4.2	0.002	0.668	1.497
Low birth weight (no*)	−11.3	−18.9 to −3.7	0.004	0.664	1.505
Siblings (no*)	−5.0	−10.0 to 0.3	0.051	0.519	1.927
Psychological and/or drug treatment (no*)	−0.2	−3.8 to 3.5	0.929	0.971	1.029

* Reference category.

AIMS: Alberta Infant Motor Scale; 95%CI: 95% confidence interval; *t:* tolerance; VIF: variance inflation factor.

Table 4 presents the mean difference in motor development assessed by the BSID-III among risk factors after cluster analysis. Our results showed that children whose only risk factor was cesarean delivery had a mean difference of 2.9 points (95%CI: 0.1 to 5.7) less than babies without risk factors. Babies with cesarean delivery and low birth weight as risk factors had a mean difference of 14.8 points (95%CI: 5.7 to 23.9) less than children without risk factors. Children with cesarean delivery and prematurity as risk factors had a mean difference of 13.1 points (95%CI: 5.6 to 20.6) less than babies without risk factors. Finally, children who had cesarean delivery, low birth weight, and prematurity as risk factors had a mean difference of 18.3 points (95%CI: 12.0 to 24.5) less than babies without risk factors.

**Table 4:** Cluster analysis using Tukey’s test with exposure variables associated with motor development using the BSID-III.

Risk factors*	Motor development at three months of age (BSID-III)		

	Mean difference	95%CI	p-value
Elective cesarean section	−2.9	−5.7 to −0.1	0.042
Cesarean and LBW	−14.8	−24.0 to −5.7	< 0.001
Cesarean and prematurity	−13.1	−20.6 to −5.6	< 0.001
Cesarean, LBW, and prematurity	−18.3	−24.5 to 12.0	< 0.001

* “No risk factor” as a reference category.

BSID-III: Bayley-III Scale of Infant and Toddler Development; LBW: low birth weight; 95%CI: 95% confidence interval.

We also performed a multivariate linear regression analysis considering the variables with p < 0.20 in the bivariate analysis (maternal age, socioeconomic status, siblings, and first pregnancy) and the cluster variable. After the adjustment, the cluster variable was the only one still associated with motor development with β −0.07 (95%CI: −0.08 to −0.05; p < 0.001). All VIF values were below 4 and tolerance was above 0.25, which ensures that multicollinearity is not a problem.

## DISCUSSION

Our results showed that the means for motor development in three-month-old children were lower among babies born by cesarean delivery, prematurely, and with low birth weight, and who had siblings. We also found that children with three risk factors (cesarean delivery, low birth weight, and prematurity) had a difference of almost 20 points less in mean motor development (BSID-III) compared with babies without any risk factors.

Regarding the type of delivery, other studies also show an association between cesarean delivery and poorer motor development. In a study conducted by Sun et al.^30^, babies born by cesarean delivery had a greater risk of delayed fine and gross motor skills at six months of age than babies born by vaginal delivery. Similarly, Zaigham et al.^31^ showed that children born by cesarean delivery had lower fine and gross motor skills at four and 12 months of age. In this same study, the authors showed that babies born by vaginal delivery had better neurodevelopmental scores at four months compared with babies born by cesarean delivery. They also observed these differences for gross motor skills at 12 months of age. According to Cavaggioni et al.^32^, cesarean delivery provides evidence of psychological risks in fine motor skills, language expressions, and the manifestation of adaptive behavior. However, Rodrigues and Silva^33^ state that even though the motor skills of children born by cesarean delivery are inferior compared with babies born by vaginal delivery, they would still be in accordance with the typical parameters for their age. On the other hand, manipulative skills, visual language, speech skills, and personal autonomy are significantly reduced in children born by cesarean delivery^33^.

Regarding the relationship between prematurity and motor development, we can assume that a premature child is more susceptible to changes in motor development, especially in the first years of life. A study conducted in Chicago, USA, with 106 premature babies, found an association between low motor development and prematurity, both in motor development results measured by the Test of Infant Motor Performance (TIMP) at three months, and in motor development measured by the BSID-III at two years of adjusted age^34^. Other studies also found the same association at 12 months^23,35,36^ and the same results when using AIMS. According to Lefebvre et al.^37^ in their study with extremely premature children (28 weeks or less) at four, 10, and 12 months of age, motor development delay was higher at four months. In an Australian study with children born at 30 weeks or less, 53% had poor motor development at an adjusted age of 12 months^38^.

Similarly, low birth weight was associated with motor development delay. Syrengelas et al.^39^ found that premature children with low birth weight had poorer motor development compared with term children on the AIMS subscales. Premature and low birth weight babies had delayed motor development from one to 19 months of adjusted age compared with children born at term^39^. Similarly, Zhang et al.^40^ found that babies with low birth weight had a higher risk of being diagnosed with motor development delay from one to six months of age compared with babies born with normal weight.

In the cluster analysis, we also found that the more risk factors, the lower the motor development mean. However, even considering cesarean delivery as the only risk factor, the groups showed a difference in their motor development means. Therefore, we reinforce that a cesarean section is a procedure that should be performed only if necessary, in cases where the life of the mother or the baby is at risk.

The occurrence of elective cesarean sections in Brazil has increased greatly since the late 1980s. In general, in the Brazilian public health system, different professionals provide care to pregnant women during the prenatal period, and cesarean delivery is indicated upon diagnosis of complications in pregnancy or childbirth. On the other hand, private health services allow the scheduling of a cesarean delivery by the indication of the obstetrician or by choice of the pregnant woman. One of the reasons that leads pregnant women to opt for cesarean delivery is the fear of vaginal delivery, especially due to the pain they may feel, which is more intense in primiparous women. In a study conducted in Brazil, more than half of the pregnant women evaluated underwent a cesarean delivery, and almost 90% of them used private health services^41^. Therefore, since cesarean delivery is a risk factor for delayed motor development, investing in awareness policies for both pregnant women and health professionals is necessary.

Our study has limitations. We collected data about the alcohol and tobacco use during pregnancy in the second stage of the study (after delivery), thus, we can assume a memory bias. Moreover, these data may be underreported: women may understand that alcohol and tobacco use is harmful to the baby’s health and may omit their use at the time of the interview.

On the other hand, this population-based study included two scales considered the gold standard for motor development. Early assessment of motor development in babies is scarce in the literature, especially in Brazil. Adopting adequate measures for early detection of development delays in children may improve their development at subsequent ages.

## CONCLUSION

This study sought to identify the risk factors associated with poor motor development at three months of age. Our results showed an association between lower motor development and cesarean delivery, prematurity, and low birth weight. The identification of risk factors for delayed motor development may allow early interventions related to factors that can be avoided in some cases, such as those observed in this study. Thus, this study helps expand knowledge about the determinants of poor early motor development in Brazil.
